# COVID-19 pandemic, pregnancy care, perinatal outcomes in Eastern Myanmar and North-Western Thailand: a retrospective marginalised population cohort

**DOI:** 10.1186/s12884-024-06841-0

**Published:** 2024-10-02

**Authors:** Taco Jan Prins, Wanitda Watthanaworawit, Mary Ellen Gilder, Nay Win Tun, Aung Myat Min, May Phoo Naing, Chanapat Pateekhum, Woranit Thitiphatsaranan, Suradet Thinraow, Francois Nosten, Marcus J. Rijken, Michele van Vugt, Chaisiri Angkurawaranon, Rose McGready

**Affiliations:** 1 Department of Family Medicine, Faculty of Medicine, Chiang Mai University, Chiang Mai, Thailand; 2 Global Health and Chronic Conditions Research Group, Chiang Mai University, Chiang Mai, Thailand; 3 Amsterdam University Medical Centre, Department of Internal Medicine & Infectious Diseases, Research Groups: APH, GH and AII&I, Amsterdam UMC, Amsterdam, The Netherlands; 4 Shoklo Malaria Research Unit, Mahidol–Oxford Tropical Medicine Research Unit, Faculty of Tropical Medicine, Mahidol University, Mae Sot, Thailand; 5 Julius Global Health, Julius Centre for Health Sciences and Primary Care, University Medical Centre Utrecht, Utrecht University, Utrecht, The Netherlands; 6 Centre for Tropical Medicine and Global Health, Nuffield Department of Medicine, University of Oxford, Oxford, UK

**Keywords:** Covid, Antenatal care, Migrants, Perinatal outcome

## Abstract

**Background:**

The COVID-19 pandemic disrupted routine health care and antenatal and birth services globally. The Shoklo Malaria Research Unit (SMRU) based at the Thailand-Myanmar border provides cross border antenatal care (ANC) and birth services to marginalised pregnant women. The border between the countries entered lockdown in March 2020 preventing cross-border access for women from Myanmar to Thailand. SMRU adapted by opening a new clinic during the COVID-19 pandemic in Myanmar. This study explored the impact of the COVID-19 pandemic and response on access to ANC and pregnancy outcomes for marginalised pregnant women in the border regions between Thailand and Myanmar.

**Methods:**

A retrospective review of medical records of all pregnancies delivered or followed at antenatal clinics of the SMRU from 2017 to the end of 2022. Logistic regression was done to compare the odds of maternal and neonatal outcomes between women who delivered pre-COVID (2017–2019) and women who delivered in the COVID-19 pandemic (2020–2022), grouped by reported country of residence: Thailand or Myanmar.

**Results:**

Between 2017 and the end of 2022, there were 13,865 (5,576 resident in Thailand and 8,276 in Myanmar) marginalised pregnant women who followed ANC or gave birth at SMRU clinics. Outcomes of pregnancy were known for 9,748 women with an EGA ≥ 28 weeks. Unknown outcome of pregnancy among women living in Thailand did not increase during the pandemic. However, there was a high (60%) but transient increase in unknown outcome of pregnancy for women with Myanmar residence in March 2020 following border closure and decreasing back to the baseline of 20–30% after establishment of a new clinic. Non-literate women were more likely to have an unknown outcome during the pandemic. There was no statistically significant increase in known stillbirths or maternal deaths during the COVID pandemic in this population but homebirth was over represented in maternal and perinatal mortality.

**Conclusion:**

Decreasing barriers to healthcare for marginalised pregnant women on the Thailand-Myanmar border by establishment of a new clinic was possible in response to sudden border closure during the COVID-19 pandemic and most likely preventing an increase in maternal and perinatal mortality.

**Supplementary Information:**

The online version contains supplementary material available at 10.1186/s12884-024-06841-0.

## Introduction

The COVID-19 pandemic has made a large impact worldwide, with approximately 700 million cases and 7 million deaths directly related to the COVID-19 virus [1]. The pandemic disrupted routine health care pathways including antenatal care, birth and postnatal services, in hospitals and clinics. Barriers to traditional maternal healthcare models were increased by the lockdown, restricted movement, l and delay in care seeking through fear of the virus or the consequences of being infected [2–10].

Quality antenatal care (ANC) reduces maternal and neonatal morbidity and mortality, by timely interventions based on risk identification, screening for disease, treatment of pregnancy related illnesses, and health promotion [11]. Disruption of ANC and increased births without skilled birth attendants increased maternal and neonatal morbidity and mortality globally, especially in low- and middle-income countries during the COVID-19 pandemic [2, 4, 6, 12]. In Thailand and other countries interventions to avoid disruptions included visits via digital or phone technology, if this was a possibility for the hospital and women [9, 13].

Thailand was the first country to publicly declare a COVID-19 patient outside China on 13 January 2020 [14, 15]. Multiple lockdowns, border closure and restrictions, started in March 2020. Thailand’s health system response was praised as a model of pandemic control, while reporting 5 million positive tested cases and approximately 34,500 deaths [15, 16].

A large migrant population of approximately 3.9 million people live and work in Thailand, which had an estimated population of 71.5 million in 2020 [17, 18]. Of these migrants, 2.1 million are from Myanmar [17, 18]. Before COVID-19 approximately half of the migrant population was undocumented and unregistered, the proportion of which likely increased during the pandemic as registration services closed [18–21]. Low-, middle- and high-income countries reported more disruption in migrant health care than health care for non-migrants during COVID-19 [22–24]. Migrants in Thailand had increased difficulty accessing healthcare and COVID-19 vaccines during the pandemic for multiple reasons: fear of getting infected, rumors about the infection and vaccine, limited health literacy and access to accurate information in their language, severe restrictions on movements with risk of arrest and deportation, loss of daily wage, lack of legal documents, less phone ownership and no health insurance [18, 21, 25].

Myanmar people have survived political instability for the last seven decades, however the COVID-19 pandemic and coup d’etat of 1 February 2021 increased both the pressure and the barriers to access basic healthcare on an overburdened and under-resourced health care system [26, 27]. The Association of Southeast Asian Nations (ASEAN) has long recognized the need for cross border collaborations as a crucial strategy for improving health outcomes [28]. Thailand has a long history of collaborating with Myanmar populations on its western most border seeking cross border health care services [29].

The objective of this study is to explore the impact of the COVID-19 pandemic for the marginalized pregnant women in the border regions between Thailand and Myanmar on access to ANC and birth services through rates of unknown outcome of pregnancy and adverse pregnancy outcomes compared to the pre-COVID period.

## Methods

Ethical approval for retrospective analysis of hospital records of the SMRU was provided by the Chiang Mai University (FAM-2566-09383), by the Oxford University Ethics Committee (OXTREC: 28 − 09) and the local community advisory board in Mae-Sot, Thailand (TCAB-4/1/2015).

### Study design

A retrospective review of medical records of pregnant women who followed ANC or gave birth from 2017 to 2022 in the clinics of the Shoklo Malaria Research Unit (SMRU) in border areas of North-Western Thailand or Eastern Myanmar. The STROBE guideline for observational studies was followed for this study [30]. The clinical details were extracted from electronic medical records and extraction was completed in December 2023. If any detail needed clarification the information was retrieved from paper-based records such as the partogram, which are stored in the archives of the SMRU. Individual identifiable patient data was removed.

### Setting and background

The SMRU provides ANC and delivery clinics for migrant and marginalised woman, on both sides of the Thailand-Myanmar border in the districts of Mae Ra Mat and Phob Prah in Tak Province. This fills a gap in services recognized by Thai Public Health for this marginalized population who mostly speak Burmese, Karen and other minority languages, and predominantly work in the Thailand agricultural sector [31, 32].

The SMRU trains staff with standardised curriculums for midwifery, basic obstetric ultrasound, and the Advanced Life Support in Obstetrics (ALSO) course developed by the American Academy of Family Physicians. Services include basic outpatient and inpatient care, antenatal clinics five days per week, on site ultrasonography, delivery room, a special care baby unit for premature and unwell neonates, and basic laboratory services on site by locally trained staff [33–36]. Hence the components of comprehensive obstetric and newborn care (CEmOc) including services for normal pregnancy and childbirth, administration of antibiotics for infection, administration of antihypertensive and anticonvulsant medication, essential newborn care, manual removal of placenta, assisted vaginal delivery and blood transfusion, can all be provided [37]. Major surgery (e.g. caesarean section) requires referral with a 24 h standby car. In such cases the woman is referred to the nearest local Thai or Myanmar public hospital (15 min to one hour drive, depending on the location of the clinic). Before the COVID-19 pandemic, pregnant women from both sides of the border visited ANC and peripartum care at the SMRU clinics on the Thai side of the border (clinics are based near the riverside which forms the border). During COVID-19, the border between Thailand and Myanmar abruptly closed at the end of March 2020 till January 2023, increasing the barriers to reach the clinics for pregnant women (and SMRU staff) on the Myanmar side of the border. To decrease the barriers for the women living in Myanmar, SMRU supported SMRU midwifery and medical staff to provide antenatal care and delivery services in a local general medical clinic run by the Karen Ethnic Health Organization Consortium April-May 2020, in what was expected to be a temporary measure. As the period of border closure due to COVID-19 was prolonged, a Myanmar doctor trained in obstetrics was employed to support care in the Myanmar clinic in November 2020.

Before the pandemic, women were seen every 2–4 of weeks if possible. During the pandemic, the number of ANC visits was reduced to a minimum of 8 to minimize the risk of COVID-19 infection for pregnant women and staff. Phone consultation was not an option because of the lack of phone ownership among marginalised women. On admission to the labour ward at SMRU clinics women were tested for COVID-19 with a PCR test (when available) as a consistent surveillance measure and to protect staff, or when they had symptoms of Covid during ANC, from October 2020 (when it became available with external funding in this population) till the end of 2022. During the COVID-19 pandemic because of COVID-19 infection and isolation or quarantine requirements in Thailand (which were mostly applied in the SMRU in Myanmar) there was less staff available at some periods compared to before the COVID-19 pandemic. Staff with confirmed COVID-19 infection did not work and could return to work in 7–14 days later depending on their wellbeing.

### Participants

All women who registered to ANC at SMRU clinics between 2017 and the end of 2022 were included, including those lost to follow-up who were classified as unknown outcome of pregnancy. Analysis of pregnancy outcomes were included for singletons only if the estimated gestational age (EGA) at birth was 28 weeks or more. An EGA of 28 weeks is considered to be the lower limit of viability in this setting, due to an extremely low chance of survival (1%) before 28 weeks [38].

### COVID-19 testing

COVID-19 infection was confirmed by real-time reverse transcription polymerase chain reaction (RT-PCR), including Sansure Novel Coronavirus (2019-nCoV) Nucleic Acid Diagnostic kit (detects N and ORF1ab genes) and/or Xpert Xpress SAR-CoV-2 (detects E and N2 genes). The tests were performed and the results were interpreted according to the manufacturers’ instructions and guidelines from the Ministry of Public Health, Thailand.

### Variables

Women were subdivided in two groups by self-reported residence: living in Thailand or Myanmar. Gravidity was the number of pregnancies a woman had including the current pregnancy; parity was the number of neonates a woman had previously delivered (alive or stillborn). Maternal education is not routinely asked and while literacy is, it overestimates true health literacy [39]. A women was defined literate if she answered that she was able to read. The EGA of the pregnant women was based on quality-controlled ultrasound assessment with the Dubowitz gestational age assessment of the neonate as a second line estimate for late ANC attendance (EGA ≥ 28 weeks). The outcome of pregnancy of a woman was declared as unknown if details about the birth were not known. The date for women with an unknown birth outcome was set to one week after their last ANC-visit. The percentage of unknown outcome was calculated by the number of unknown outcomes divided by known and unknown outcomes per month. Hypertension in pregnancy was defined as systolic blood pressure ≥ 140 mmHg or diastolic blood pressure ≥ 90 mmHg. Pre-eclampsia was defined as hypertension in pregnancy in combination with protein in the urine or with danger signs such as headache and blurred visions. Postpartum haemorrhage was defined as blood loss ≥ 500 ml after birth. The place of birth was given only for singletons as multiple pregnancies could have multiple places of birth [40]. Place of birth was recorded as SMRU clinic, hospital (public or private), home or other clinic. Occasionally women gave birth on the way to clinic and these were counted as home as there was no skilled attendant present. Stillbirth rate was defined as number of neonates born with no signs of life at an EGA ≥ 28 weeks per 1000 total births. Maternal death included women who died during pregnancy and up to 6 weeks postpartum in women with a known outcome of pregnancy with an EGA ≥ 28 weeks. The number of COVID-19 infections in Thailand and Myanmar was sourced from the WHO resource database of COVID-19 infections [41, 42]. The total reported COVID-19 cases of the Thailand and Myanmar, which have a population approximately 70 million and 54 million inhabitants respectively [43], reflects the health systems difference of both countries, with the under-resourced Myanmar health system reporting less COVID-19 cases but approximately reporting the same waves in period of times as Thailand.

### Statistical analysis

Data were analyzed using R (version 4.2.1) for Windows. Continuous data were described using the mean, standard deviation minimum, and maximum. Categorical data were summarized using frequency and proportions. Chi-squared test was used to compare overall data between resident in Thailand and Myanmar. The level of significance was set as *p* < 0.05. Comparisons of antenatal care and birth outcomes are for two periods: pre-COVID-19 (Jan-2017 to Dec-2019) and during the COVID-19 pandemic (Jan-2020 to Dec-2022); however for analysis of the number of COVID-19 infections the period is Oct-2020 to Dec-2022 for birth outcomes as this is the period when COVID-19 tests were available. Multivariable logistic regression was performed to compare obstetric complications between pre-COVID-19 women and COVID-19 pandemic women, adjusting for the potential confounders: maternal age, parity, smoking, literacy and multiple gestation. The year 2020 was chosen because the first case reported was in January 2020. Adjusted Odds ratios were also calculated for perinatal outcomes between women by timeframes: pre-pandemic or during the pandemic, and for women who had a known outcome between 2020 and 2022. This was done only for perinatal and neonatal outcomes for singletons due to the low rate of twins in this population as reported previously [40].

## Results

**Fig. 1:**
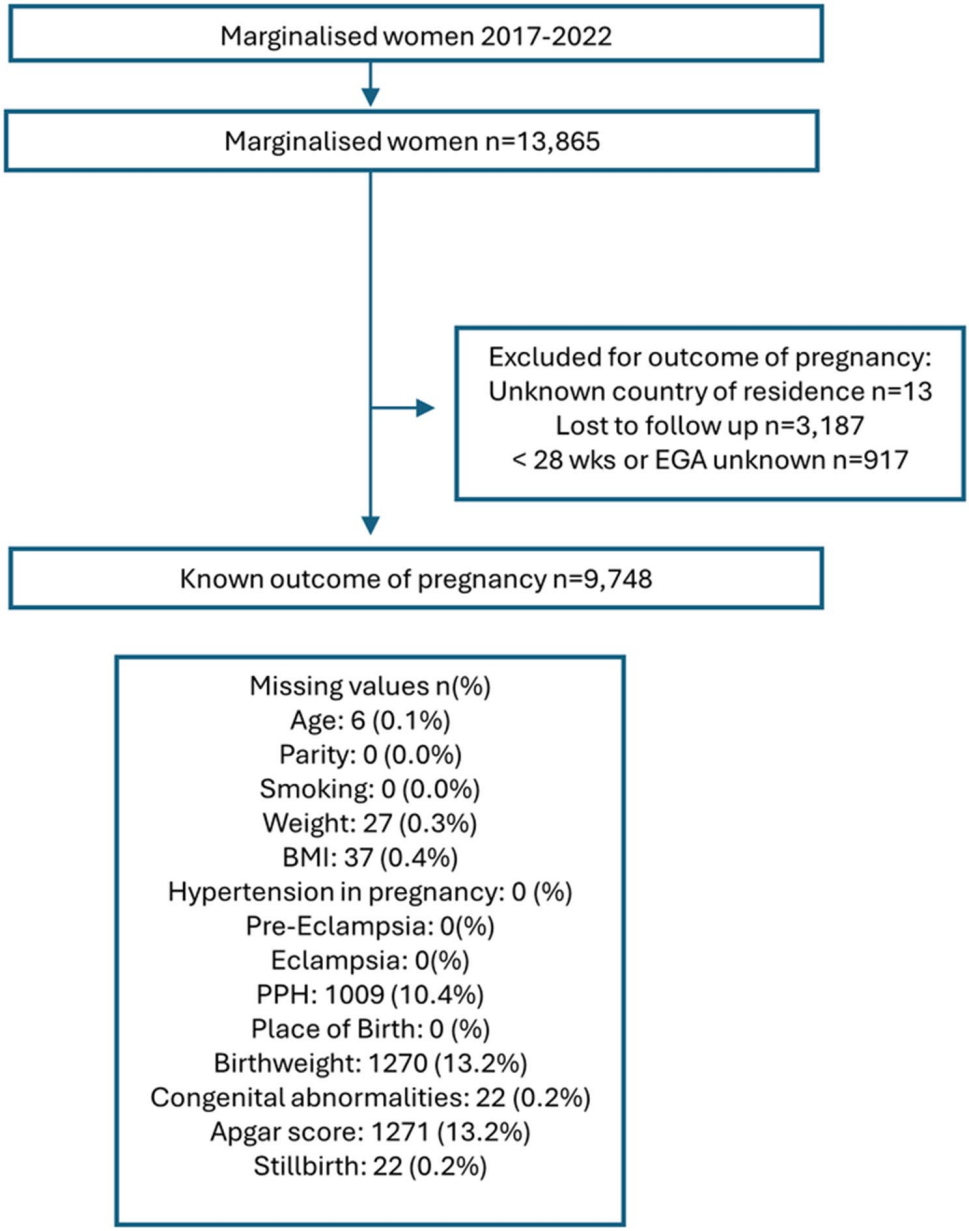
Flow chart of data available and missing values

Between 2017 and the end of 2022, there were in total 13,865 pregnant women who gave birth or followed ANC care at the clinics of the SMRU (Fig. 1). Of these 40.2% (*n* = 5,576) gave their residence as Thailand and 59.7% (*n* = 8,276) as Myanmar. There were 70.3% (*n* = 9,748) of women with a known outcome of pregnancy with an EGA ≥ 28 weeks; 1.0% (*n* = 96)) were multiple pregnancies and 99.0% (*n* = 9,652) were singleton pregnancies; with 40.0% (3,910/9,748) reporting residence as Thailand and 59.9% (5,838/9,748) as Myanmar.

### Number of COVID-19 infections and COVID-19 related maternal mortality

**Fig. 2:**
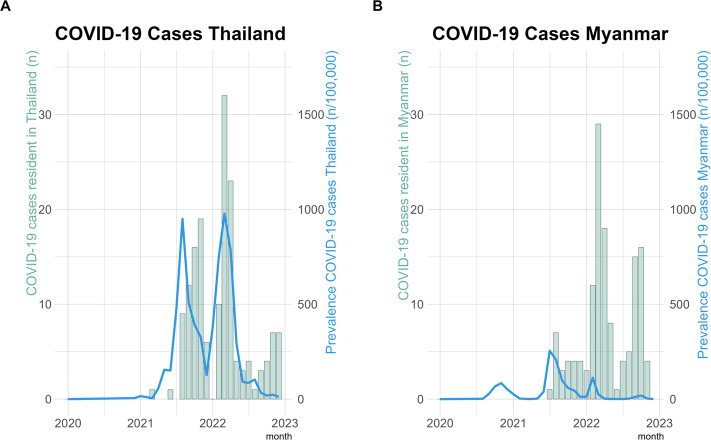
COVID-19 cases per month in clinics and total cases COVID-19 in Thailand and Myanmar. **A** COVID-19 cases in the clinics in Thailand and COVID-19 cases in Thailand, **B** COVID-19 cases in clinics in Myanmar and COVID-19 cases in Myanmar. Screening by nasopharyngeal swab and polymerase chain reaction was done for all patients starting October 2020 with exceptions due to extenuating circumstances

Based on availability of COVID-19 testing capacity there were 2,717 births in SMRU clinics of whom 72.7% (1,975/2,717) were tested for COVID-19 at least once in pregnancy during the pandemic (October 2020 to the end of 2022). COVID-19 infection was detected in 12% (151/1,252) of pregnant women in Thailand and 19% (138/723) in Myanmar. The timing of positive COVID-19 infections of pregnant women followed a similar pandemic wave pattern to the national populations (Fig. 2A and B) though testing in Myanmar was limited after mid-2021 (Fig. 1B). In this marginalised population testing was available only from October 2020. From March 2020 till September 2020 there was no COVID-19 infection wave in Thailand and a first small wave, peaking September-October 2020 in Myanmar. Although there was nearly no COVID-19 infections from March 2020 till September 2020 there were severe restrictions on movements of migrants and the border was closed from early 2020.

There was one confirmed COVID-19 mortality in this population: a 39 year old non-smoker, gravida 5 parity 4 (all normal vaginal deliveries) with gestational diabetes, no COVID vaccination and no other significant medical history. She complaining of 2 days fever and developed acute respiratory distress with a confirmed COVID-19 infection at 35 weeks EGA, and was transferred from the SMRU clinic but died in Myawaddy Hospital, Myanmar.

### Outcome of pregnancy

**Fig. 3:**
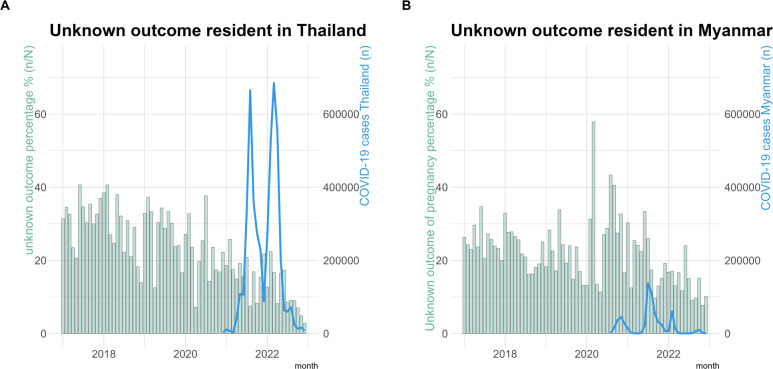
Percentage unkown outcome of pregnancy per month with COVID-19 cases in Thailand and Myanmar **A** Percentage unknown outcome of pregnancy per month of women resident in Thailand with COVID-19 cases Thailand, **B** Percentage unknown outcome of pregnancy per month of women resident in Myanmar with COVID-19 cases Myanmar

The overall proportion of unknown outcome of pregnancy by reported residence, pre-pandemic (2017–2019) and during the pandemic (2020–2022) was: 23.7% (1,323/5,576) [95%CI 22.6–24.9] in Thailand and 22.5% (1,864/8,276) [95%CI 21.6–23.4] in Myanmar (*P* = 0.099). Unknown outcome of pregnancy affected a higher proportion of women resident in Myanmar than Thailand (Fig. 3A and B). The peak was at 38% in Thailand in July 2020 and not out of range of monthly unknown outcomes from the previous 2 years. While a peak approaching 60% in Myanmar occurred in March 2020 after closure of the border. With the opening of the new clinic in April-May 2020 the proportion of unknown outcome of pregnancy started to decrease. The proportion of literate women with an unknown pregnancy outcome was significantly lower than known pregnancy outcome during the pandemic: 66.0% (872/1,321) and 75.4% (4,012/5,320), *P* < 0.05; contrary to no significant difference before the pandemic: 63.9% (1,193/1,866) and 65.5% (3,501/5,344), *P* = 0.23.

### Morbidity during pregnancy

While there were minor differences in baseline characteristics of women resident in Thailand and Myanmar, the proportion of women smoking in pregnancy decreased during the pandemic compared to pre-pandemic timeframes, irrespective of reported residence) (Table 1). Similarly, the percentage of obstetric complications such as gestational hypertension, pre-eclampsia and PPH remained similar for women with a Thailand and Myanmar residence (Table 2).

**Table 1:** Maternal characteristics resident in Thailand and Myanmar

Maternal characteristics	Resident in Thailand 2017–2019 (*n* = 1,995) Pre-pandemic	Resident in Thailand 2020–2022 (*n* = 1,915) During pandemic	Resident in Myanmar 2017–2019 (*n* = 2,896) Pre-pandemic	Resident in Myanmar 2020–2022 (*n* = 2,942) During pandemic
Age (years) at first antenatal visit, Mean (SD) [min-max]	26.0 (6.9) [13–47]	25.8 (7.0) [14–46]	26.3 (6.7) [14–52]	25.6 (6.8) [13–50]
Gravidity, median [min-max]	2 [1–14]	2 [1–11]	2 [1–13]	2 [1–14]
Parity, median [min-max]	1 [0–9]	1 [0–12]	1 [0–11]	1 [0–10]
Nulliparous, % (n)	40.7 (812/1,995)	42.1 (806/1,915)	37.6 (1,088/2,896)	45.5 (1,340/2,942)
Literacy, % (n)	66.2 (1,320/1,995)	73.0 (1,397/1,915)	65.6 (1,899/2,896)	77.4 (2,277/2,942)
Smoker, % (n)	6.2 (124/1,995)	3.4 (65/1,915)	11.4 (331/2,896)	5.7 (168/2,942)
ANC in First trimester, % (n)	31.9 (636/1995)	33.0 (631/1,915)	36.0 (1,044/2,896)	32.2 (946/2,942)
First trimester; mean (SD) [min-max]				
Weight (kg)	48.3 (8.5) [30–89]	50.6 (9.9) [31–102]	49.7 (9.0) [31–107]	51.3 (9.7) [27–120]
BMI (kg/m^2^)	20.8 (3.4) [13.6–36.1]	21.7 (3.9) [13.6–43.2]	21.5 (3.6) [14.5–45.1]	22.0 (3.7) [14.0-43.3]
Multiple gestation, n (%)	0.6 (11/1,995)	0.8 (15/1,915)	1.3 (39/2,896)	1.1 (31/2,942)

**Table 2:** Obstetric complications women resident in Thailand and Myanmar in 2017–2019 and 2020–2022

Maternal complication outcome	Resident in Thailand % (*n*/*N*)		adjOR^a^ (95%CI)	Resident in Myanmar % (*n*/*N*)		adjOR^a^ (95%CI)
Years	2017–2019 Pre-pandemic	2020–2022 During pandemic		2017–2019 Pre-pandemic	2020–2022 During pandemic	
Estimated Gestational Age (weeks) at birth (SD); mean [min-max]	39.1 (1.5) [28–43]	39.0 (1.7) [28–44]	NA	38.9 (1.8) [28–43]	38.8 (1.8) [28–44]	NA
Preterm labour						
28 + 0 to < 32 weeks	0.6 (12/1,995)	0.9 (18/1,915)	1.71 (0.81–3.63)	1.1(31/2,896)	1.3 (39/2,942)	1.30 (0.80–2.10)
32 + 0 to < 37 weeks	5.5 (109/1,995)	6.4 (122/1,915)	1.19 (0.91–1.55)	7.4 (214/2,896)	7.2 (213/2,942)	1.03 (0.84–1.26)
Term labour ≥ 37 weeks	93.9(1,874/1,995)	92.7(1,775/1,915)	reference	91.5 (2,651/2,896)	91.4 (2,690/2,942)	reference
Gestational Hypertension	7.7 (154/1,995)	9.5 (182/1,915)	1.25 (0.99–1.57)	8.6 (248/2,896)	8.9 (263/2,942)	1.09 (0.91–1.31)
Pre-eclampsia	2.8 (56/1,995)	2.8 (53/1,915)	0.97 (0.66–1.43)	2.6 (75/2,896)	2.4 (72/2,942)	1.02 (0.73–1.43)
Eclampsia	0.3 (6/1,995)	0.3 (6/1,915)	0.94 (0.29–3.04)	0.6 (17/2,896)	0.3 (10/2,942)	0.54 (0.24–1.17)
Postpartum haemorrhage	7.0 (128/1,835)	8.1 (138/1,714)	1.16 (0.90–1.50)	5.7 (145/2,543)	6.1 (161/2,653)	1.05 (0.83–1.33)

^a^ adjusted for Age, Parity, Smoking, Literacy and Multiple Gestation

### Place of birth

**Fig. 4:**
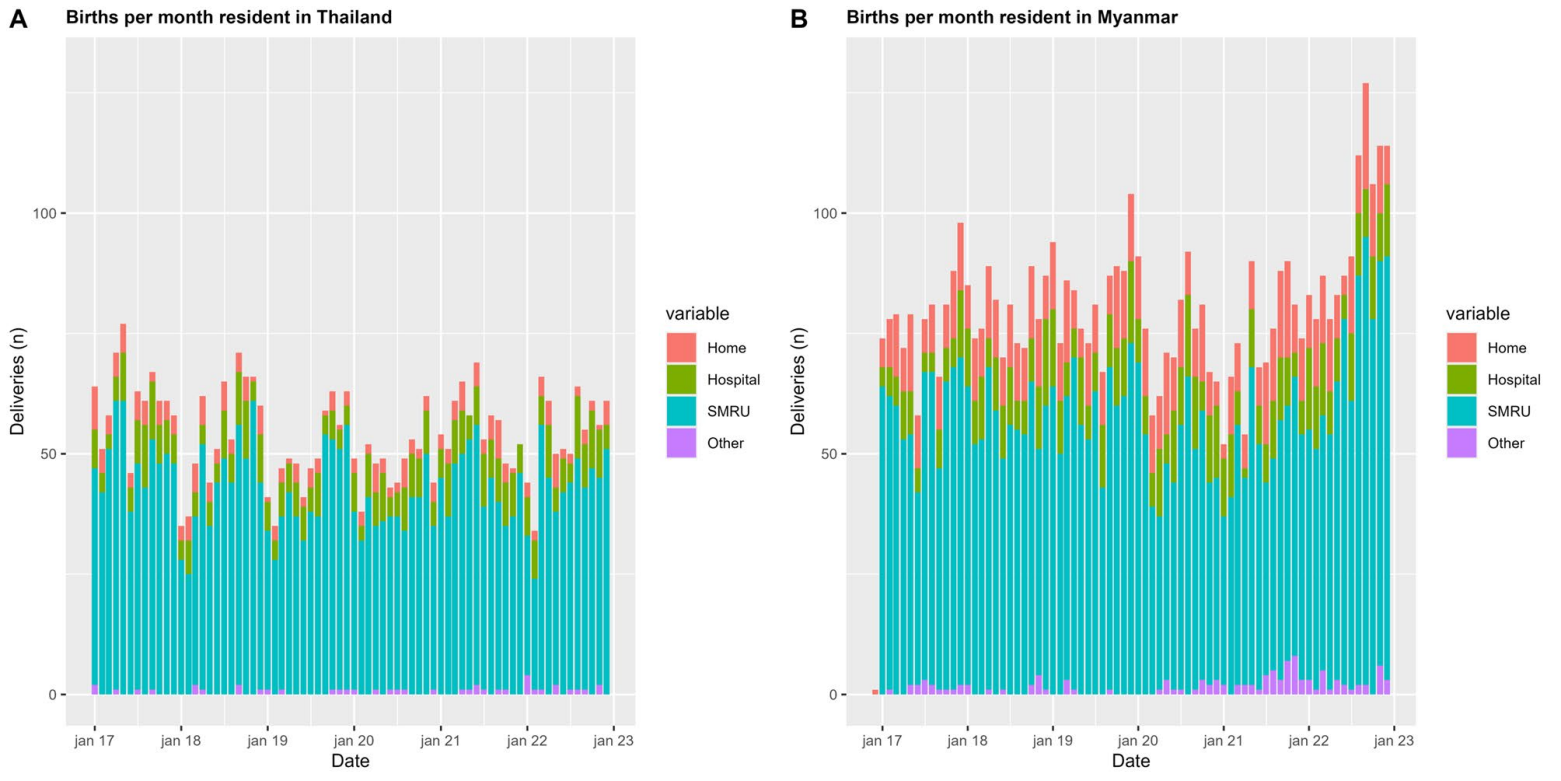
Place of birth women per month in Thailand and Myanmar. **A** Place of birth women resident in Thailand, **B** place of birth women resident in Myanmar

SMRU, rather than a public government or private hospital or home, continued to provide the most frequent site of birth in this population throughout pre and during COVID-19 pandemic although it decreased significantly from pre- to during the COVID-19 pandemic (Table 3; Fig. 4A and B). This was complemented by a slight increase births in hospital and other clinics. The lowest number of clinic births occurred in January 2022 in Thailand coinciding with Thailand’s second wave; while the lowest number in Myanmar occurred in April 2020 (border closure) and again in Feb 2021 (the month of the *coup d’etat*) without relation to Myanmar’s reported COVID-19 waves (Fig. 4A and B). Home birth was more common in Myanmar 15.2% (445/2,911) [95% CI 14.0-16.6] than Thailand residents 6.5% (124/1,900) [95% CI 5.5–7.7], (*P* < 0.001) in 2020–2022 (Table 3). Stillbirth as expected was more common in the referral hospitals which care for complicated cases. However amongst women who gave birth at home there were 5 stillbirths (5/440) in Myanmar residents compared to none with a Thailand residence (0/121) in the COVID-19 pandemic (S1 table).

**Table 3:** Place of birth singleton women resident in Thailand and Myanmar in 2017–2019 and 2020–2022

Years	Resident in Thailand % (*n*/*N*)		OR (95%CI)	Resident in Myanmar % (*n*/*N*)		OR (95%CI)
	2017–2019 Pre-pandemic	2020–2022 During pandemic		2017–2019 Pre-pandemic	2020–2022 During pandemic	
SMRU	80.1 (1,590/1,984)	77.5 (1,472/1,900)	Reference	72.9 (2,084/2,857)	68.9 (2,005/2,911)	Reference
Hospital	12.0 (239/1,984)	14.6 (278/1,900)	1.26 (1.04–1.51)	11.4 (326/2,857)	13.1 (382/2,911)	1.22 (1.04–1.43)
Home or on the way to clinic	7.3 (144/1,984)	6.5 (124/1,900)	0.93 (0.72–1.19)	15.1 (430/2,857)	15.2 (445/2,911)	1.08 (0.93–1.24)
Other clinic	0.6 (11/1,984)	1.4 (26/1,900)	2.55 (1.26–5.19)	0.6 (17/2,857)	2.7 (79/2,911)	4.83 (2.85–8.17)

Univariate comparisons: proportion of clinic births decreased from pre- to during pandemic periods: *p* = 0.041 Thailand, *p* < 0.001 Myanmar

### Perinatal outcomes

There was no clinically significant change in birthweight during and before the COVID-19 pandemic and neither an increase in congenital abnormalities (Table 4). For women resident only in Thailand the COVID-19 pandemic was associated with an increase in the proportion of Apgar scores lower than 7 at 5 min, adjOR 2.50 (1.17–5.81).

**Table 4:** Perinatal and neonatal outcomes for women resident in Thailand and Myanmar in 2017–2019 and 2020–2022

Years	Resident in Thailand % (*n*/*N*)		adjOR (95%CI)	Resident in Myanmar % (*n*/*N*)		adjOR (95%CI)
	2017–2019 Pre-pandemic	2020–2022 During-pandemic		2017–2019 Pre-pandemic	2020–2022 During-pandemic	
Birthweight (g), Mean (SD) [Min – Max]^a^	2938 (425) [600-4,340]	2970 (436) [560–4340]	NA	2964 (457) [520–5580]	2968 (486) [570–5700]	NA
Congenital abnormality % (n/N)^a, c^	2.0 (39/1,983)	2.1 (40/1,893)	1.13 (0.72–1.78)	2.2 (62/2,852)	1.6 (46/2,902)	0.71 (0.48–1.05)
APGAR < 7 at 5 min % (n/N)^a,b,c^	0.5 (9/1,811)	1.2 (21/1,741)	2.50 (1.17–5.81)	1.0 (23/2,416)	0.8 (18/2,350)	0.85 (0.45–1.58)
Stillbirth rate^a,c^	5 per 1000 (10/1,983)	6 per 1000 (12/1,893)	1.47 (0.62–3.54)	9 per 1000 (26/2,852)	10 per 1000 (28/2,902)	1.15 (0.66–1.99)
Maternal Death per livebirths	0 (0/1,995)	0 (0/1,915)	NA	0.2 (4/2,896)	0.03 (1/2,942)	NA

^a^ Only singletons included

^b^ Only live births

^c^ Adjusted for maternal age, parity, literacy, gestational hypertension, pre-eclampsia, eclampsia, smoking

### Stillbirth

**Fig. 5:**
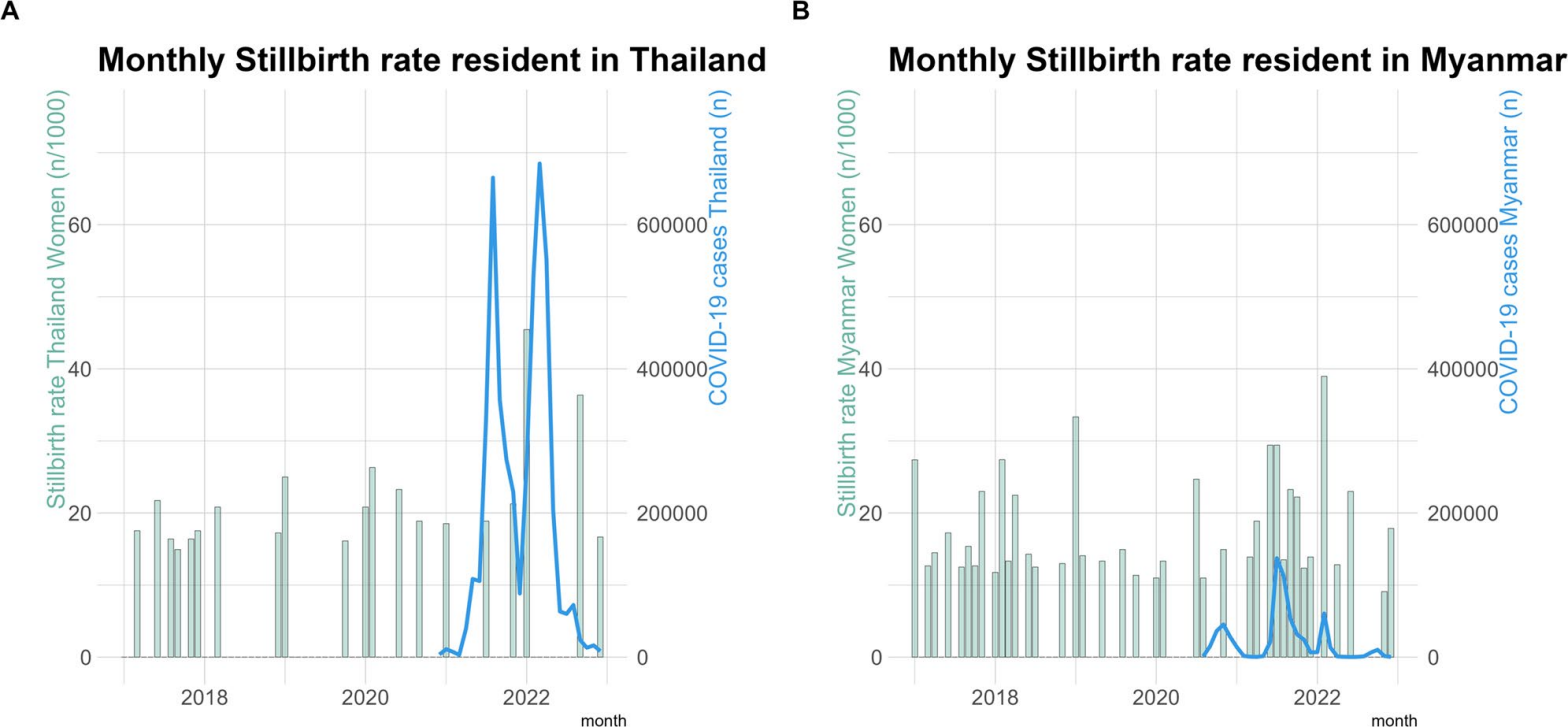
Stillbirth rate per month with covid cases in Thailand and Myanmar. **A** Stillbirth rate of women resident in Thailand with COVID-19 cases Thailand, **B** Stillbirth rate of women resident in Myanmar with COVID-19 cases Thailand

The COVID-19 pandemic was not associated with a significantly increased risk of stillbirth compared to pre-COVID-19 for women resident in Thailand, adjOR 1.47 (95% CI 0.62–3.54) or Myanmar, adjOR 1.15 (95% CI 0.66–1.99) (Table 4). The highest monthly stillbirth rates in women resident in both Thailand (January 2022) and Myanmar (February 2022) coincided with the early phase of the second major COVID-19 wave for the region in early 2022 (Fig. 5a). Similarly, in women resident in Myanmar the peak was during a COVID-19 wave in February 2022 (Fig. 5a and b). When stillbirth rates were highest in January 2022 in women resident in Thailand, there were 2 stillbirths related to pre-eclampsia, and both had only two ANC visits during pregnancy, with no visit in the last month. In February 2022 there were 3 stillbirths in women resident in Myanmar. One fetal death was of unknown etiology where the mother had more than 8 ANC visits, and two fetal deaths were associated with pre-eclampsia in women who had less than 4 ANC visits during pregnancy and the first visit in late second trimester so pre-eclampsia prevention with provision calcium and aspirin supplements was not possible.

### Maternal mortality

There were no known maternal deaths in women resident in Thailand in the population with an EGA of at least 28 weeks and a known outcome of pregnancy. In women resident in Myanmar there were 4 maternal deaths in 2017–2019 and 1 in 2020–2022. All of the deaths in 2017–2019 were during home births and causes were PPH, eclampsia, puerperal sepsis and one death was unknown. In 2020–2022 the causes of death was a uterine rupture from an attempted home birth.

## Discussion

This retrospective study shows that decreasing the barrier to healthcare for vulnerable populations during the COVID-19 Pandemic was possible by opening a new clinic for ANC and child birth on the Thailand-Myanmar border. The closure of the border increased the number of unknown outcome of pregnancy temporarily, by making it more difficult for vulnerable people to access healthcare. The number of unknown outcome decreased back to the baseline level when SMRU opened a new clinic for ANC and childbirth. With the continuation of ANC and care for childbirth there was no increase of morbidity and mortality in the available data with known outcomes.

Before and during the COVID-19 pandemic, SMRU clinics continued to serve the population for care surrounding pregnancy and birth. The percentage of clinic births decreased during the pandemic, regardless of residence without a significant increase in home births, and a statistically increased rate (small proportional increase) of hospital and ‘Other clinic’ (low proportion overall) births. This is in contrast to reports in other low resource settings: studies in Kenya and Rwanda showing a decrease of institutional births [44, 45]. This outcome was possible because of SMRU’s strong cohort of local staff motivated and trained to a degree where they could establish a new safe antenatal and birth service in Myanmar in a midwife led clinic. Movement of essential equipment was possible and the team were later strengthened by the presence of a Myanmar doctor, rather than support by phone.

This study did not detect an increase in pre-eclampsia or eclampsia during the COVID-19 period although an increase may have been expected as COVID-19 in pregnancy is associated with increased risk of hypertensive diseases in pregnancy [46]. This study did not look into the association of an infection with COVID-19 and hypertensive disorders. This was not done because the numbers of COVID-19 infections were small in this cohort and most of the COVID-19 tests were done when the patient was admitted for birth and not during pregnancy. However the routine data on smoking in pregnancy showed a marked decrease in smoking during the COVID-19 period compare to the pre-pandemic period, which is consistent with a recent systematic review including 58,052 non-pregnant participants [47]. In a prospective cohort study in Greece in 283 women surveyed from Jan to May 2020, tobacco consumption decreased in the perinatal period compared to the preconception period potentially from COVID-related restrictions and fear of potential illness [48]. This is in contrast to a UK based study of 252 pregnant women referred to cessation programs that reported no change in cessation rates and demand for antenatal smoking cessation services, before and during the COVID-19 pandemic [49].

An important difference between country of residence was the proportion of homebirths with a two fold higher rate in women resident in Myanmar. This reflects the situation in the countries. As reported in the Myanmar Demographic and Health Survey 2015–16 two thirds of births occur at home, with only a 60% presence of skilled attendants [50]. In Thailand, Tak Province this proportion is much lower in some districts, but is still reported to be as high as 11% and associated with geographical difficulties to reach a clinic [51]. Notably, almost all maternal deaths and a high proportion of stillbirths in this cohort occurred during home births without skilled birth attendants.

This study shows no differences in birthweight and congenital abnormities during and before the pandemic. Viral and non-viral stressors such as anxiety increases the risk on low birthweight and congenital abnormalities [52, 53]. There is conflicting evidence on the association on COVID-19 infection during pregnancy and outcomes including birthweight and congenital abnormalities; in addition studies where it is reported were not designed to test this association [52–55]. There was an increase in Apgar scores lower than 7 in Thailand residents during the pandemic. Staff shortage and increased barriers to reach healthcare could explain the increase. This is similar to a study in Malawi reporting an increase in birth asphyxia, due to staff shortage [56] while this was not reported in high income countries [57].

The stillbirth rate differed by country of residence, with women resident in Myanmar having a higher stillbirth rate. Thailand’s nationally reported stillbirth rate is 5.45 per 1000 births, while the rate in Myanmar is 14.54 per 1000 births [58]. The barriers to reach care resulting in homebirth without skilled birth attendants is the most likely cause of the higher stillbirth rate in Myanmar [59]. The overall stillbirth rate did not change significantly increase during the COVID-19 pandemic. This is in line with a meta-analysis which shows no significant increase in stillbirths pre and during the pandemic in high and low resource countries [60]. Contrary to this are studies from Nepal and Israel which reported an increase in stillbirth during the pandemic [61, 62]. Increases in stillbirth are thought to be because of difficulty accessing the health facility or fear for the virus or consequences of being infected [2, 62].

There was no significant increase or decrease of known maternal deaths in this population but the baseline for women resident in Myanmar was already high. This contrasts with studies that reported significantly more maternal deaths [2, 57, 63]. SMRU’s efforts to decrease the barrier for women to reach the clinic to support skilled attendance at birth likely reduced the risk of mortality. However, maternal deaths in this population are most likely underreported before and during the COVID-19 pandemic and is a recognized limitation of this analysis. There was a statistically significant increase in unknown pregnancy outcome but this was sporadic and short live and very likely minimized by the emergency opening of a new clinic.

Barriers to reach healthcare increased worldwide during the pandemic and is considered one of the contributing factors to the observed excess of mortality globally [64]. Not surprisingly in low-income countries, where access to healthcare is already difficult, maternal and perinatal mortality increased more in comparison to high income countries [57, 64]. While our study shows that literacy was an enabler to reaching care at the clinic during the pandemic but not before the pandemic we were unable to explore reasons for this dropout from care e.g. rumours about catching COVID-19 at the clinic, or fear of arrest. Lessons from COVID-19 on pandemic preparedness includes protection of vulnerable groups e.g. pregnant women, refugees, but does not specify non-literate [65]. Telemedicine used in high-income countries as a solution for reduced face-to-face care is not a solution for migrants and people in most low-resource settings [57]. At the Thailand-Myanmar border there is a lack of phone ownership, unstable/absent network in rural areas, or changing phone numbers with short-term sim-card deals. Balancing the direct and indirect effects of the pandemic and the restrictions is difficult, nearly impossible, in a situation that is new for everyone [57, 65]. To reduce maternal and neonatal mortality and morbidity in the future, including during pandemics, there should be a focus on decreasing barriers for vulnerable populations to essential healthcare.

The most important limitation of this study is the retrospective and observational aspect of the data. Due to the restrictions and border closure it only reflects the pregnant women who registered to antenatal care in this population, and the number of undocumented migrants in this border setting remains a labile estimate. As SMRU has provided care in the area since 1997 and has always recorded unknown outcome of pregnancy, the increase during the pandemic is based on robust data. There is a risk of bias for example there are sporadic increases in unknown outcome, with up to 60% in one month in Myanmar when the border was closed. Hence the number of obstetric complications including stillborn and maternal deaths are likely underestimated. As the aim of this study is to focus on the utilization of the ANC and pregnancy outcomes of this population it still provides a rare view of the disruption of care for a largely invisible and undocumented marginalized pregnant population. This study did not look into the socioeconomic or employment status of the women which could influence the obstetric complications and perinatal outcomes, as this information has not been routinely collected. During the COVID-19 pandemic the challenges of migrants and vulnerable people worldwide increased with less social security e.g. job insecurity, which influences their quality of life. This and the increasing barriers to access healthcare, could influence the obstetric complications and perinatal outcomes [66, 67]. Pregnancy and childbirth can become life-threatening with collapse of health care due to pandemics and conflicts or in the situation of migration and undocumented status [68]. The unstable and fragile nature of health at the Thailand Myanmar border has resulted in a 30 year focus on having skilled local staff who can provide a safe level of basic health care during pregnancy and childbirth and this review of a rare dataset suggests there is a benefit to this. Borders are frequently seen as a sight of safety in conflict zones globally. The strength of the study is that it shows the utilisation of ANC and pregnancy outcomes of a marginalised population during the COVID-19 pandemic and demonstrates the importance of organizational flexibility to respond rapidly to reduce barriers to healthcare.

## Conclusion

Establishing a new clinic to provide antenatal care and birth service to marginalized women in response to the sudden border closure during the COVID-19 pandemic reduced barriers to care and most likely prevented an increase in maternal and perinatal mortality. Rapid adaption was possible with a workforce largely trained and living in an environment of neglected and unstable health care in Myanmar.

## Electronic supplementary material

Below is the link to the electronic supplementary material.

Supplementary Material 1

## Data Availability

Data of the SMRU cannot be shared publicly because this is a population of undocumented refugees and migrants and we do not have their permission to share their data. Data are available from the Mahidol-Oxford Research Unit Institutional data access committee upon reasonable request from researchers who meet the criteria for access to confidential data (contact Rita Chanviriyavuth, email rita@tropmedres.ac).

## Abbreviations

PPH: Postpartum hemorrhage
ANC: Antenatal care
SMRU: Shoklo Malaria Research Unit
EGA: Estimated gestational age
