# Supplementary material for: COVID-19 pandemic, pregnancy care, perinatal outcomes in Eastern Myanmar and North-Western Thailand: a retrospective marginalised population cohort
Source: BMC Pregnancy Childbirth. 2024 Oct 2;24:637. doi: 10.1186/s12884-024-06841-0 (PMC11448279; doi:10.1186/s12884-024-06841-0)
Supplement: Supplementary file 1 — Supplementary Material 1 [file 12884_2024_6841_MOESM1_ESM.docx]

Supplementary table 1: Stillbirth rate per place of birth and residence, Thailand or Myanmar in 2017-2019 and 2020-2022

|  | Resident in Thailand | | Resident in Myanmar | |
| --- | --- | --- | --- | --- |
| Years | 2017-2019  Pre-pandemic | 2020-2022  During-pandemic | 2017-2019  Pre-pandemic | 2020-2022  During-pandemic |
| SMRU | 4 per 1000 (6/1,589) | 5 per 1000 (7/1472) | 7 per 1000 (14/2,083) | 7 per 1000 (14/2,005) |
| Hospital | 17 per 1000 (4/239) | 18 per 1000 (5/277) | 28 per 1000 (9/324) | 22 per 1000 (8/378) |
| Home/on the way to SMRU/clinic | 0 per 1000 (4/144) | 0 per 1000 (0/121) | 7 per 1000 (3/429) | 11 per 1000 (5/440) |
| Other clinic | 0 per 1000 (0/11) | 0 per 1000 (0/23) | 0 per 1000 (0/16) | 13 per 1000 (1/79) |

Only singletons included

Data are rate per 1,000 (n/N)
